# Supplementary material for: Single-cell transcriptomics reveal transcriptional programs underlying male and female cell fate during Plasmodium falciparum gametocytogenesis
Source: Nat Commun. 2024 Aug 26;15:7177. doi: 10.1038/s41467-024-51201-3 (PMC11347709; doi:10.1038/s41467-024-51201-3)
Supplement: Supplementary file 1 — Supplementary Information [file 41467_2024_51201_MOESM1_ESM.pdf]

Supplementary Figure 1

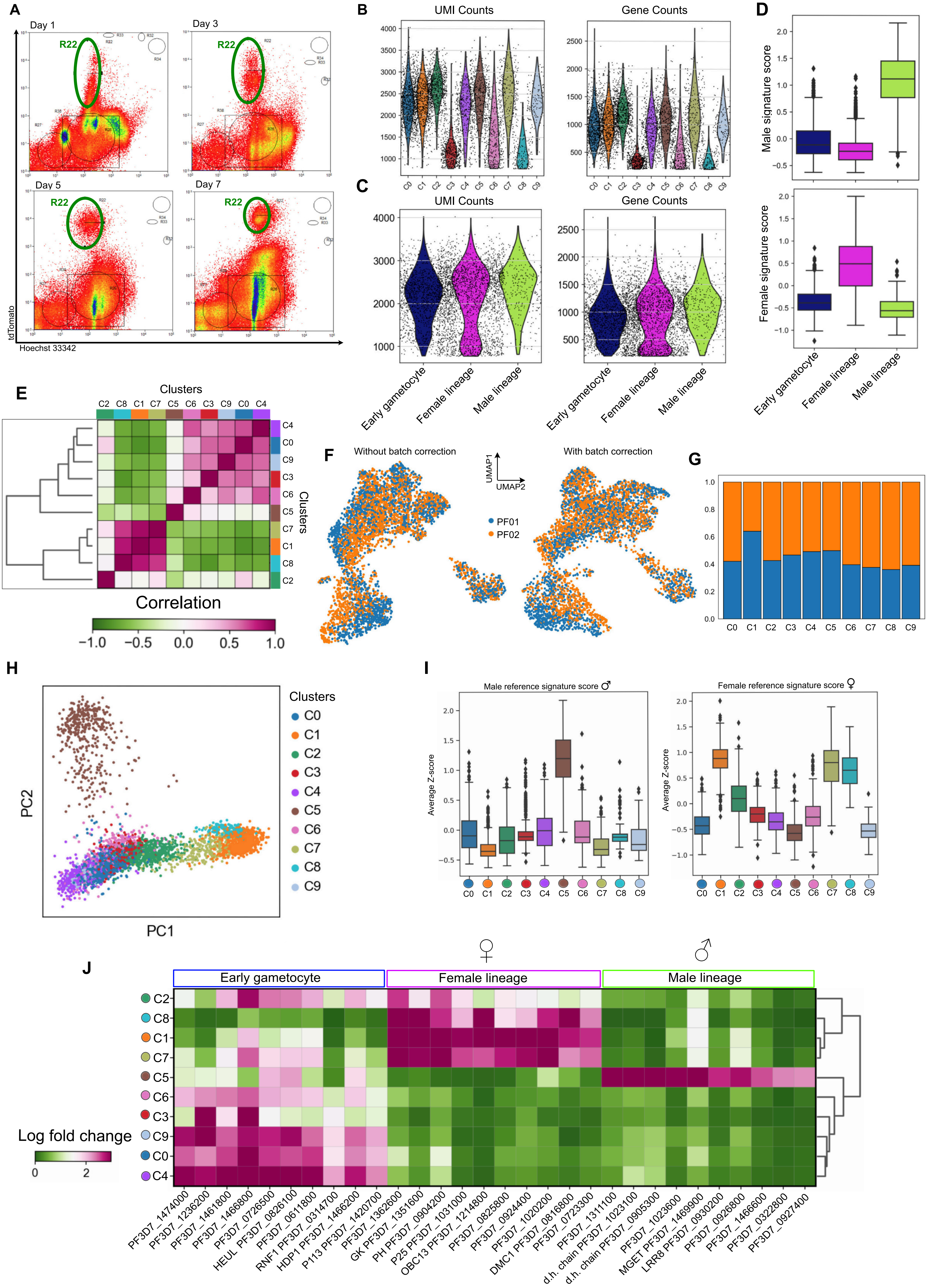

Supplementary Figure 1: scRNA-seq data quality control

A. FACS scatter plots showing the gating strategy used for cell sorting of post-induction sexual stage time points for enrichment of the gametocyte populations. Sorting gate R22 (highlighted in green) of tdTomato side (SSC) and Hoechst live cell stain 33342 forward (FSC) scatter with 400 events at each time point. B. Quality control measures of the scRNA-seq data, number of transcripts (UMI_counts), and the number of genes detected (n_genes) visualized based on the UMAP cluster identities. C. Cell class (DDRTree-based) quality control, number of transcripts (UMI_counts), and number of genes detected (n_genes) across annotated cell class. D. Male gene signature score estimated based on cell class (top panel). Female gene signature score estimated based on cell class (bottom panel). E. Correlation plot depicts the global transcriptome similarities and differences across different clusters using Pearson's correlation. The color scale indicates the correlation strength between different identified clusters; green indicates low correlation, red indicates high correlation. F. UMAP dimensionality reduction plots depicting the distribution of each library sample in a batch uncorrected dataset (left panel) and a BBKNN batch corrected dataset (right panel). The library samples include PF01 (n = 2140 cells) and PF02 (n = 2415 cells). G. Bar plot showing the sample library composition across clusters. H. Gametocyte cell population clusters projected on a PCA, based on the selection of highly informative genes for downstream analysis. The PCA is overlaid with a color code that represents the UMAP clusters. I. Box plot showing the average Z-score estimation of reference sex signature genes across UMAP clusters for male (left graph), and female (right graph) gametocytes. J. Heatmap depicting the top 10 expressed, representative marker genes identified across DDRTree cell class annotations (Wilcoxon-rank test, adj. *p value* < 0.05) and mapped to cell population clusters. The color scale intensity represents log fold changes in expression, the column identifiers include cell classes mapped to the UMAP clusters.

Supplementary Figure 2

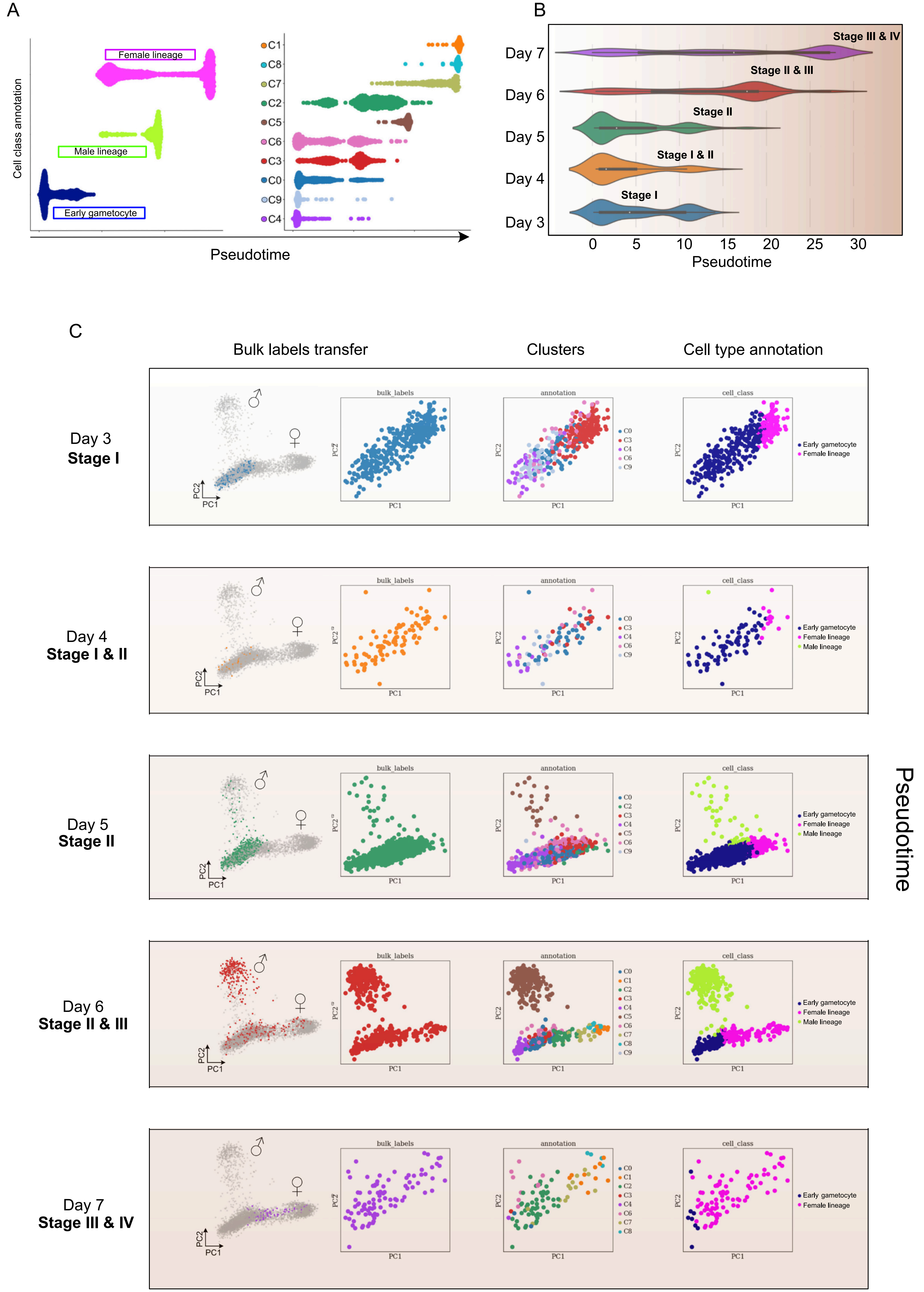

Supplementary Figure 2: Deconvolution-based spearman's rank correlation of bulk gametocyte transcriptome data from van Biljon et al 2019 *vs* Mohammed et al., 2023

A. Gametocytes cell populations aligned along the pseudotime axis. The left panel represents DDRTree annotated cell classes, and the right panel includes identified cell population clusters ordered along the pseudotime axis. B. Deconvolution of bulk gametocyte transcriptome data from van Biljon *et al* 2019 projected onto the single cell transcriptome PCA from Mohammed et al (days post-induction), compared using Spearman's correlation coefficient. C. PCA projections of bulk gametocyte transcriptomes, including cell type labels from van Biljon et al., 2019, aligned to pre-estimated pseudotime bins from the Mohammed et al study. Transferred bulk labels depict overall cell type from the bulk data projected over the PCA, based on the scRNA-seq data from this study. Annotations include cells from this study's UMAP clustering. Cell type annotation corresponds to cells from this study's single-cell transcriptome DDRTree cell class.

Supplementary Figure 3

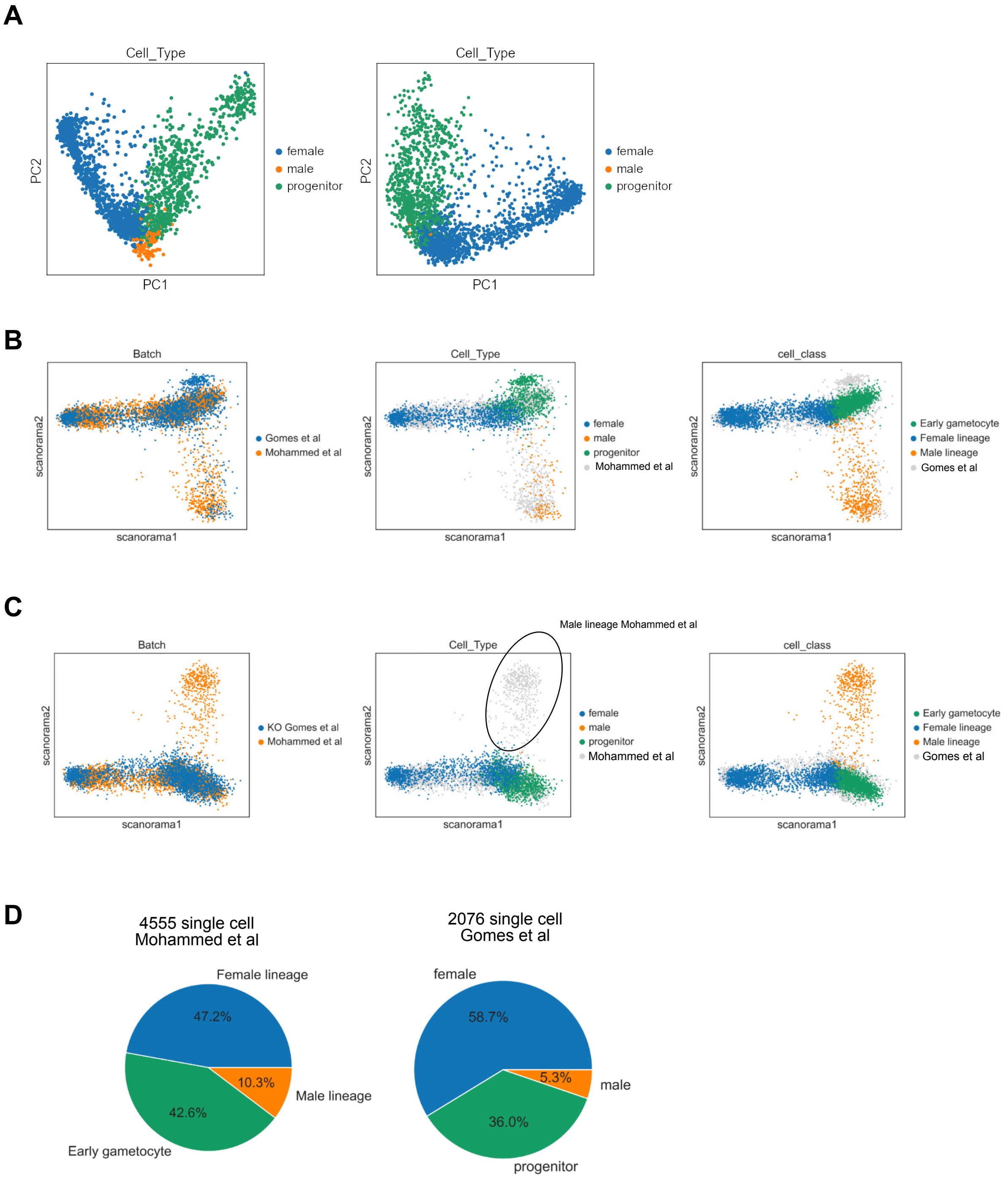

Supplementary Figure 3: Data integration with the single cell data from Gomes et al 2022

A. PCA plots including single cell data from the Gomes et al study, obtained from the Zenodo repository (Accession No. 7211710). scRNA-seq data from WT parasites (left panel) and scRNA-seq data from the *md1* knockout parasite cell line (right panel). The PCA plots are colored based on the Gomes et al cell type annotation. B. PCA plots, including the integration of single-cell data from WT parasites from the Gomes et al study with single-cell data from this study, using the Scanorama package (left panel). The same PCA plot highlights the Gomes et al single cell data in color, over this study's dataset in gray (middle panel) and with the colors for cell type delineations from this study (right panel) C. PCA plots comparing the scRNA-seq data from the md1 knockout parasite cell line from Gomes et al with the scRNA-seq data from this study. Integration of the two datasets distinguished by color (left panel). The same PCA plot highlights the Gomes et al single cell data in color over our dataset in gray (middle panel) and with the reversed colors for cell type delineations from this study (right panel). D. Pie charts depicting the total number of cells that passed the quality control between the two studies, including proportions of early gametocytes, as well as male and female gametocytes. Gametocyte stage identifiers correspond to the definitions from each respective study.

Supplementary Figure 4

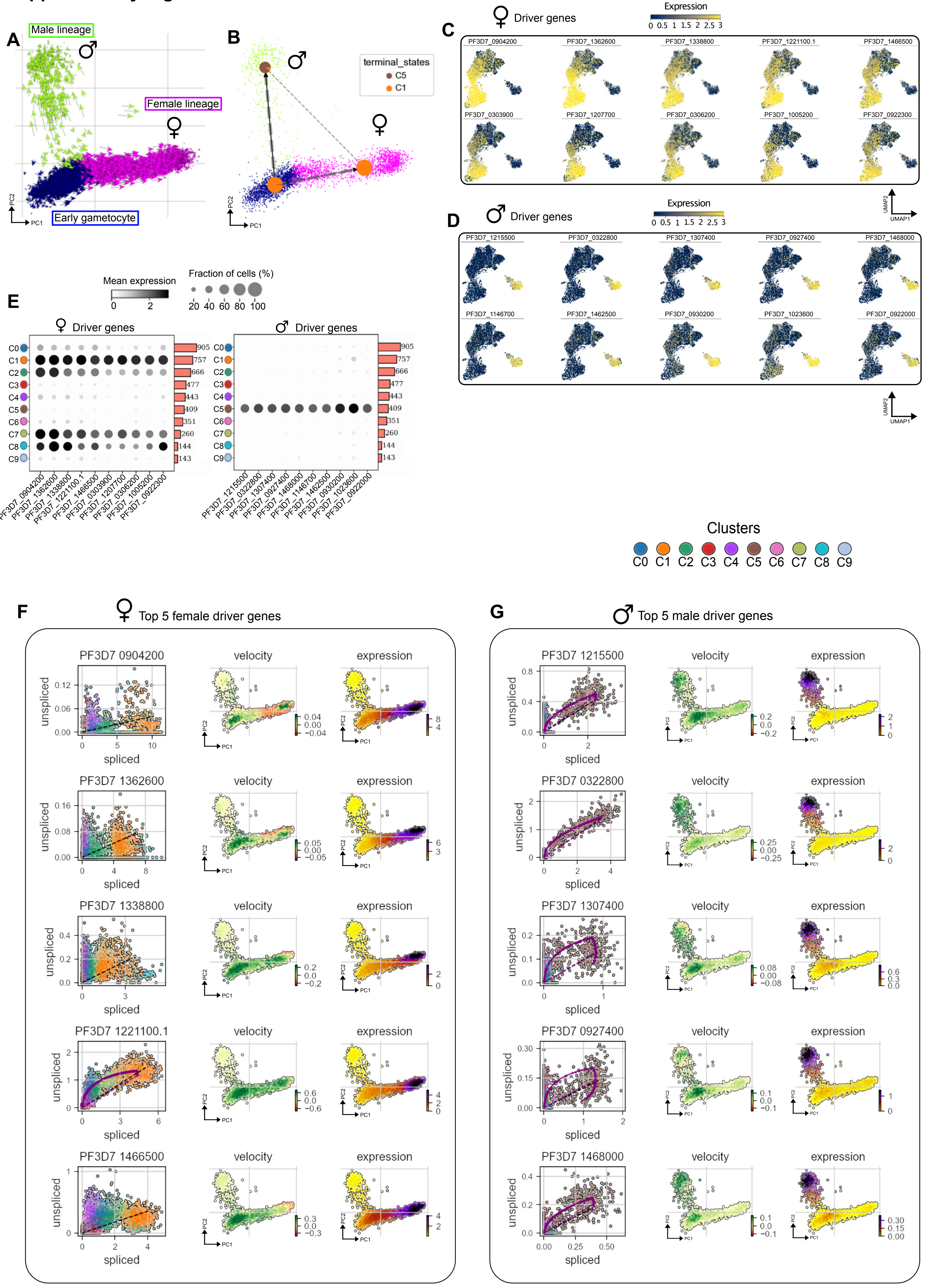

Supplementary Figure 4: Demonstration of the splicing kinetics of the top putative lineage-specific driver genes inferred by single-cell RNA velocity and cell fate lineage tracing

A. RNA velocity estimates projected across the PCA, to visualize the transcriptional dynamics of the female and male lineages overlaid by cell class annotations obtained from cell-cell similarity trajectory inference. B. Cell fate probability calculated and projected, using PAGA on PCA, reflecting the cell class annotation transcriptomes similarity towards either terminal state (C5 for mature male and C1 for mature female gametocytes). C. Top 10 female lineage putative driver gene expression profiles projected on the UMAP. D. Top 10 male lineage putative driver gene expression profiles projected on the UMAP. The color scale indicates the level of expression value with low expression (dark blue) and high expression (yellow). E. Dot plot depicting the mean expression level and the proportion of cells that show expression of lineage-specific, putative driver genes for the female (left panel) and male (right panel) gametocytes. F and G. RNA velocity estimates of unspliced, immature transcripts vs mature, spliced transcripts of the top 5 female (F) and male (G) putative driver genes (left columns), followed by RNA velocity expression estimates (middle columns) of the top 5 identified female (F) and male (G) putative driver genes. Expression level of the mature mRNA of the same genes towards either of the two terminal states (right columns) projected across the same PCA. The color scale in the velocity measurements (middle columns) indicates a high ratio of unspliced mRNA (green) and a low ratio of unspliced mRNA (red). The color scale in the expression measurements (right columns) indicates high expression level represented by purple to black where black is the highest, and low expression is represented by red to yellow, where yellow is the lowest.

Supplementary Figure 5

Female putative driver genes

A

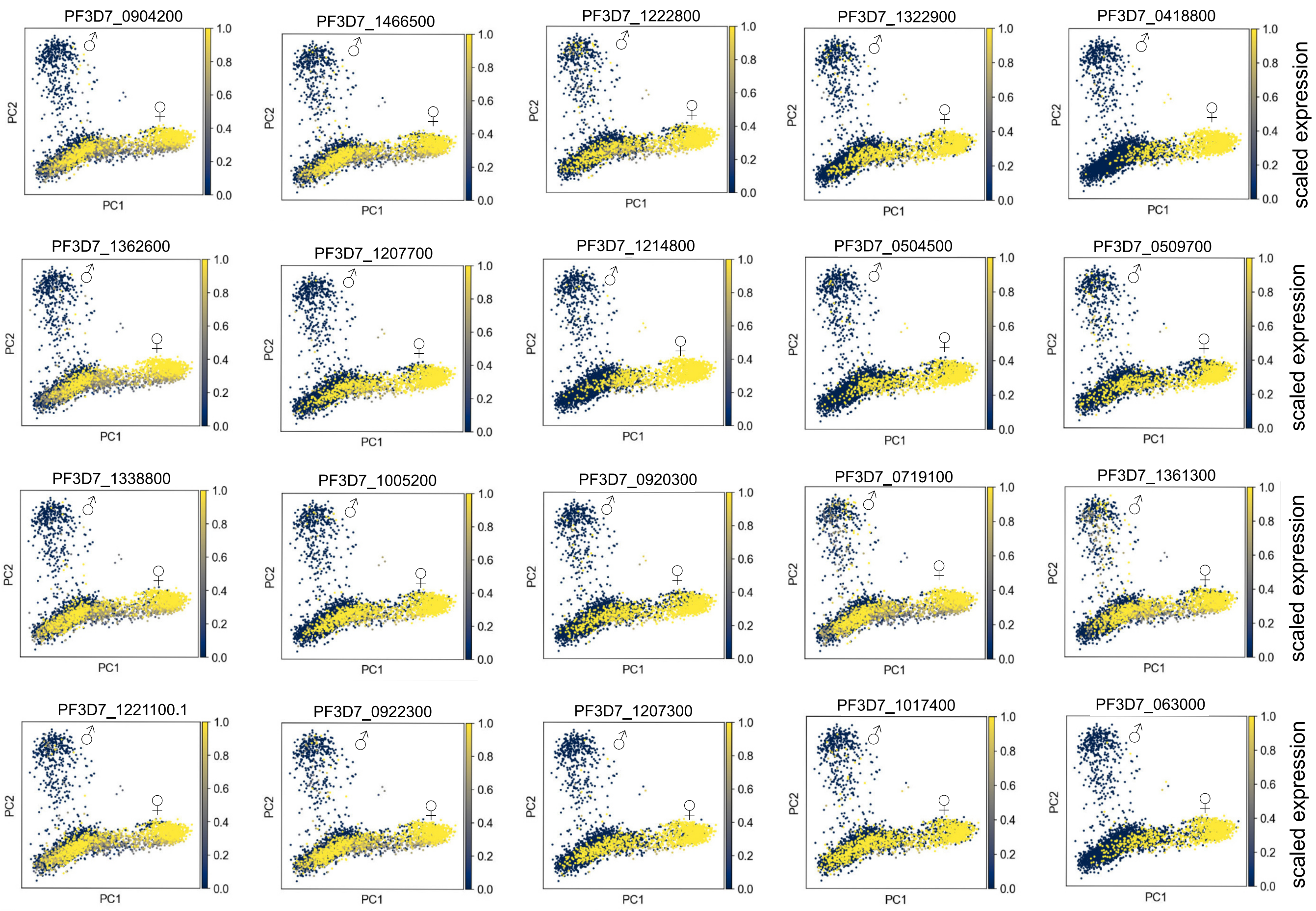

B

GENE ID	Description	GENE ID	Description
PF3D7_0904200	PH,PH domain-containing protein, putative	PF3D7_1361300	conserved protein, unknown function
PF3D7_1362600	conserved protein, unknown function	PF3D7_0630000	CPW-WPC family protein
PF3D7_1338800	conserved protein, unknown function		
PF3D7_1221100.1	conserved protein, unknown function		
PF3D7_1466500	conserved protein, unknown function		
PF3D7_1207700	41-3 protein		
PF3D7_1005200	conserved protein, unknown function		
PF3D7_0922300	conserved protein, unknown function		
PF3D7_1222800	conserved protein, unknown function		
PF3D7_1214800	OBC13, conserved protein, unknown function		
PF3D7_0920300	conserved protein, unknown function		
PF3D7_1207300	LIMP protein, putative		
PF3D7_1322900	conserved protein, unknown function		
PF3D7_0504500	MOLO1 domain-containing protein, putative		
PF3D7_0719100	ATP synthase F0 subunit a-like protein, putative		
PF3D7_1017400	HAD5, phosphomannomutase		
PF3D7_0418800	MOLO1 domain-containing protein, putative		
PF3D7_0509700	conserved protein, unknown function		

Supplementary Figure 5

Male putative driver genes

C

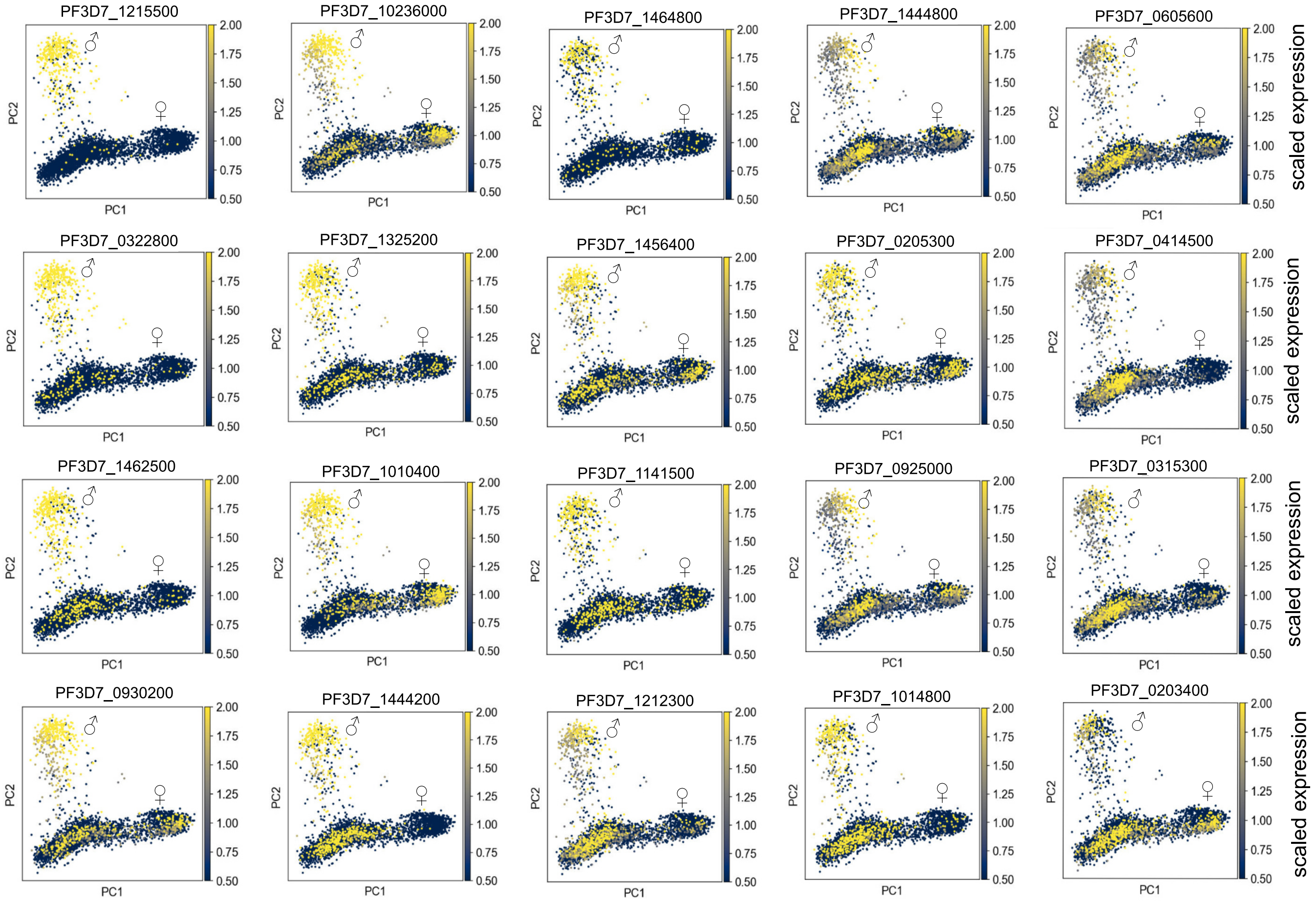

D

GENE ID	Description	GENE ID	Description
PF3D7_1215500	conserved protein, unknown function	PF3D7_0315300	conserved protein, unknown function
PF3D7_0322800	conserved protein, unknown function	PF3D7_0203400	conserved protein, unknown function
PF3D7_1462500	conserved protein, unknown function		
PF3D7_0930200	LRR8, leucine-rich repeat protein		
PF3D7_1023600	conserved protein, unknown function		
PF3D7_1325200	lactate dehydrogenase, putative		
PF3D7_1010400	MORN repeat protein, putative		
PF3D7_1444200	EF-hand calcium-binding domain-containing protein, putative		
PF3D7_1464800	EF-hand calcium-binding domain-containing protein, putative		
PF3D7_1456400	conserved protein, unknown function		
PF3D7_1141500	conserved protein, unknown function		
PF3D7_1212300	WD repeat-containing protein, putative		
PF3D7_1444800	FBPA, fructose-bisphosphate aldolase		
PF3D7_0205300	conserved protein, unknown function		
PF3D7_0925000	conserved protein, unknown function		
PF3D7_1014800	EF hand domain-containing protein, putative		
PF3D7_0605600	nucleoside diphosphate kinase, putative		
PF3D7_0414500	RNA-binding protein, putative		

Supplementary Figure 5: Visualization of gene expression among the top ranked putative female and male driver genes across the PCA

A. The top 20 female putative driver genes, selected based on the presence of sequence coverage at the 3´ exon-intron regions. Genes are ranked based on the continuous expression correlation to cluster C1 (mature female cluster). B. Panel of gene accession numbers and available descriptions from PlasmoDB. C. The top 20 male putative driver genes, selected based on the presence of sequence coverage at the 3´ exon-intron regions. Genes are ranked based on the continuous expression correlation to C5 (mature male cluster) D. Panel of gene accession numbers and available descriptions from PlasmoDB.

Supplementary Figure 6

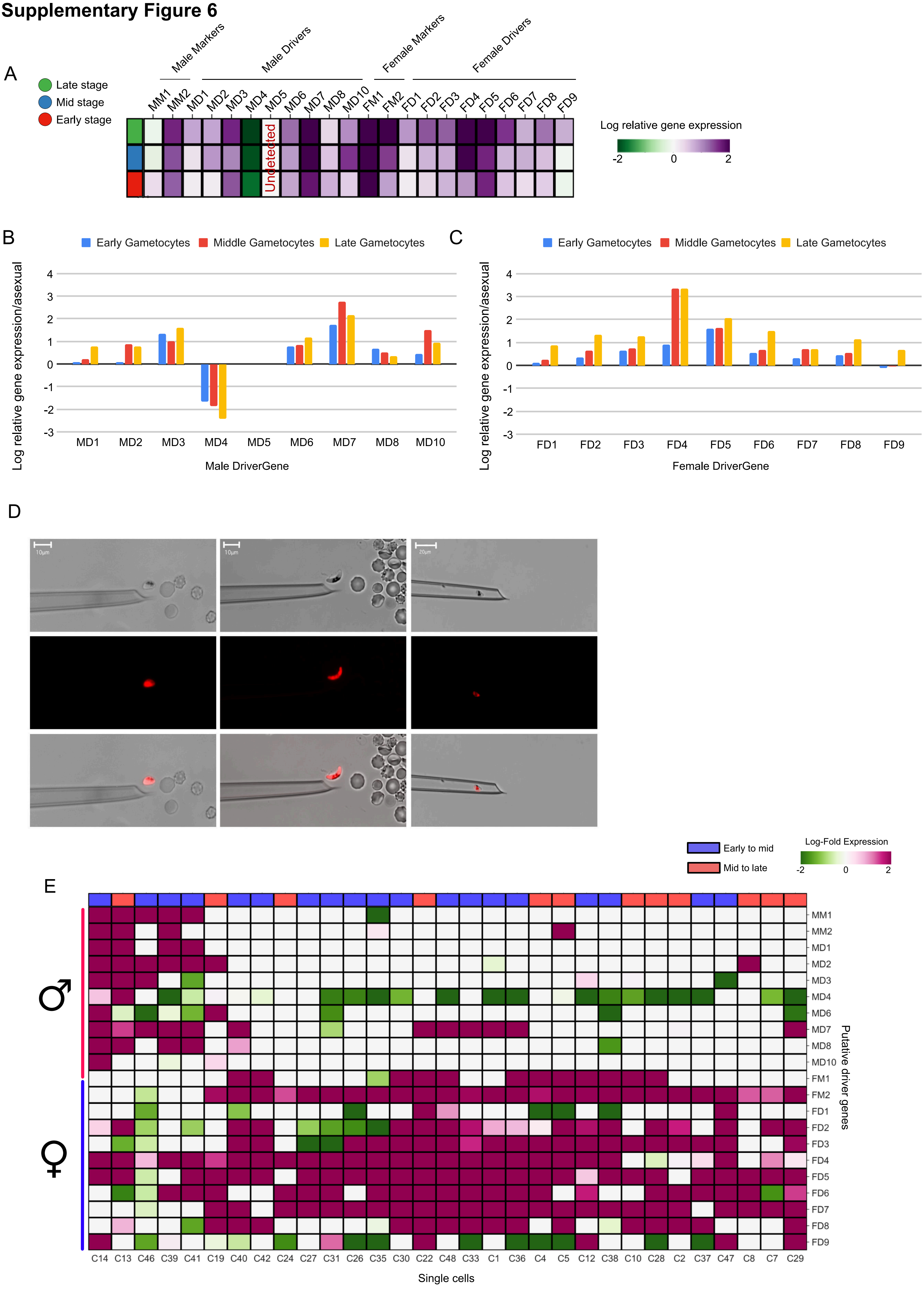

Supplementary Figure 6: Bulk and single cell rt-qPCR cross-validation of driver gene expression

A. Heatmap of the logarithmic relative fold expression of the putative male (MD) and female (FD) driver genes and previously known male (MM) and female (FM) marker genes in bulk gametocyte cultures, at different time points of development. B. Bar graph showing the log fold relative expression of top ranked, putative male driver genes in bulk cultures at different developmental stages. C. Bar graph showing the log fold relative expression of top ranked, putative female driver genes in bulk gametocyte cultures at different time points of development. D. Brightfield and fluorescent imaging depicting the microcapillary isolation of *P. falciparum* NF54 peg4-tdTomato early to mid-gametocytes (left column) and mid to late gametocytes (middle column). Individual gametocytes were subsequently deposited in 0.5 µl PBS (right column), followed by transfer to the lysis buffer. E. Heatmap of the relative log fold expression from the single-cell rt-qPCR analysis, of putative male and female driver genes and previously known markers, displayed across individually captured gametocytes.

Supplementary Figure 7

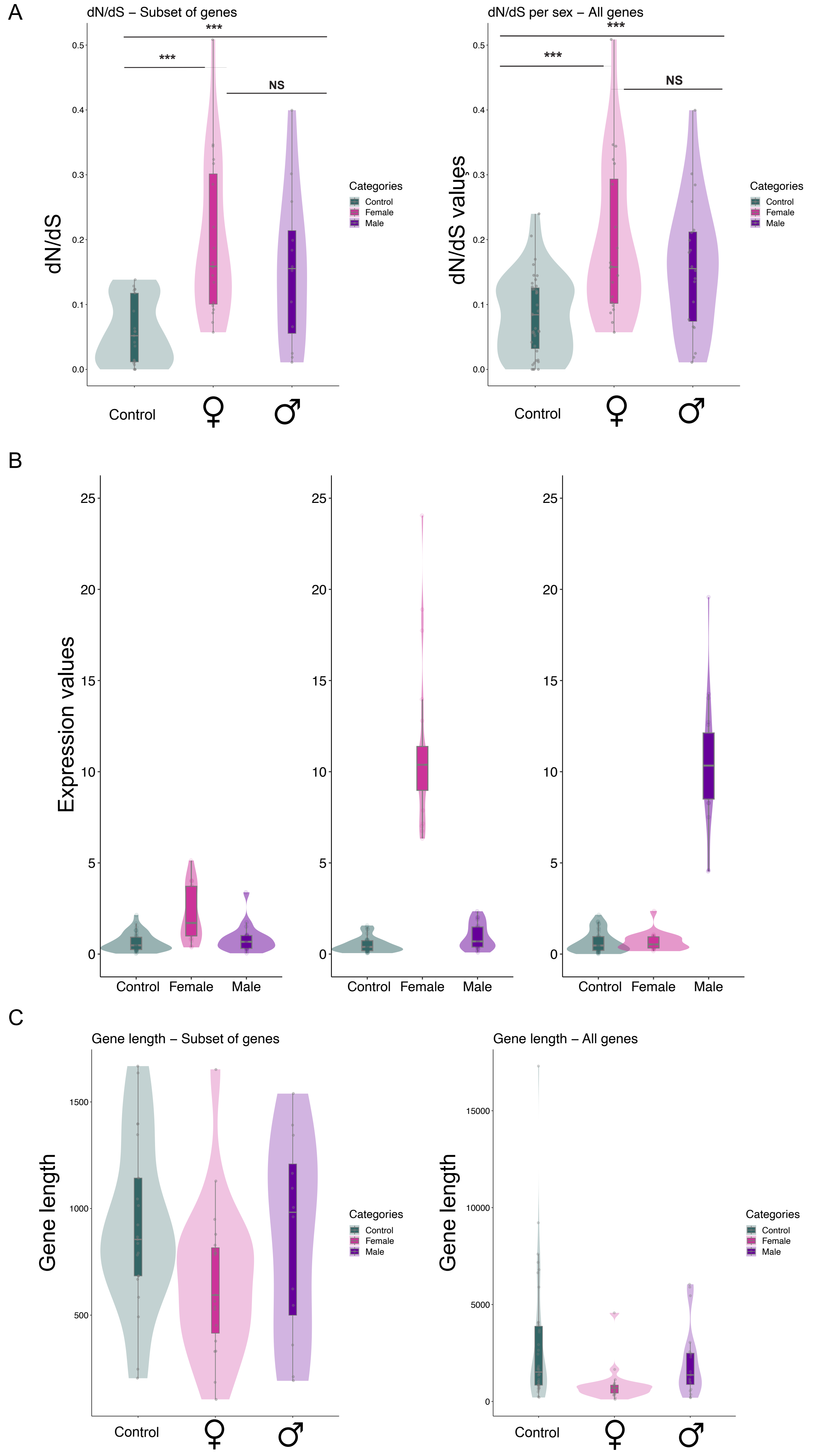

Supplementary Figure 7: Selective Pressure Analysis on Sex-Driver Genes *vs* Control Genes

A. Violin and box plots comparing *P. falciparum* and *P. gaboni* dN/dS ratios (the ratio of nonsynonymous to synonymous substitutions rates) for putative male and female driver genes compared to nuclear control genes (Kruskal-Wallis chi2=18.13, df=2, P=0.00012; Dunn post-hoc test between female and male, Z = 0.726, P=n.s.). B. Expression level analysis featuring violin and box plots, comparing the expression levels of genes in early gametocyte stages (left) with that of the female (middle) and male (right) lineages, compared to control genes (Kruskal-Wallis chi2=13.412, df=2, P < 0.01.; Dunn post-hoc test between female and male, Z = -2.700, P= =n.s.). C. Gene Length Comparison. Violin and box plots illustrating the variations in gene length among control and the putative male and female driver genes (Kruskal-Wallis chi2=20.119, df=2, P < 0.001.; Dunn post-hoc test between female and male, Z = 0.951, P= <0.001).

Supplementary Figure 8

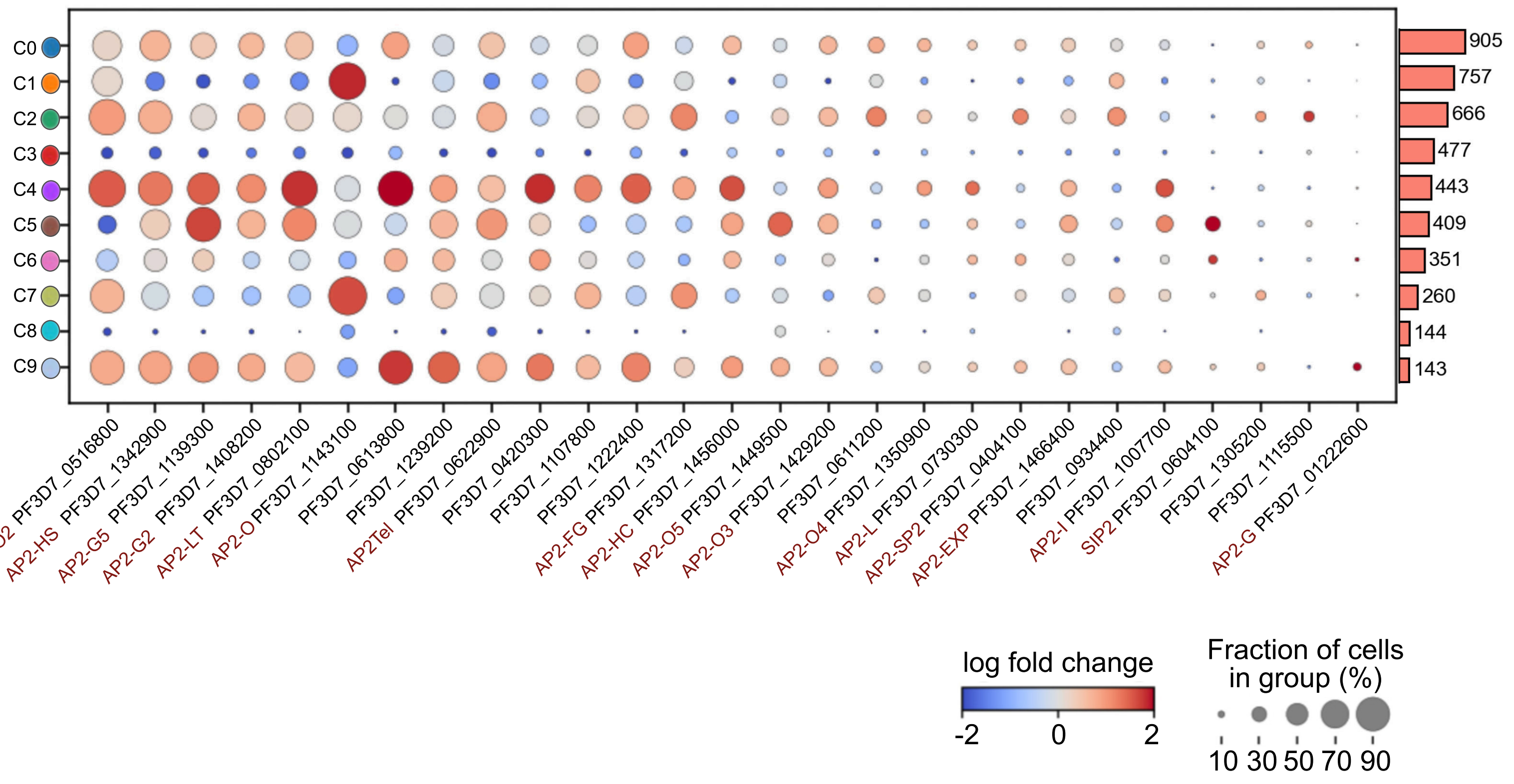

Supplementary Figure 8: Expression of ApiAP2 genes among identified gametocyte cell clusters

Expression profiles of the 27 ApiAP2 transcription factors according to cluster identities obtained using unsupervised Louvain clustering. The dot plot depicts the log fold expression of individual ApiAP2 genes as well as the proportion of cells in each cluster that expresses each ApiAP2 gene. The color code indicates changes in log fold expression, where down-regulated genes are shown in blue and up-regulated genes are shown in red. Dot sizes indicate the percentage of cells expressing each gene within the cluster. Transcriptomes from 4555 cells from two sample libraries were included in the analysis.

Supplementary Figure 9

A

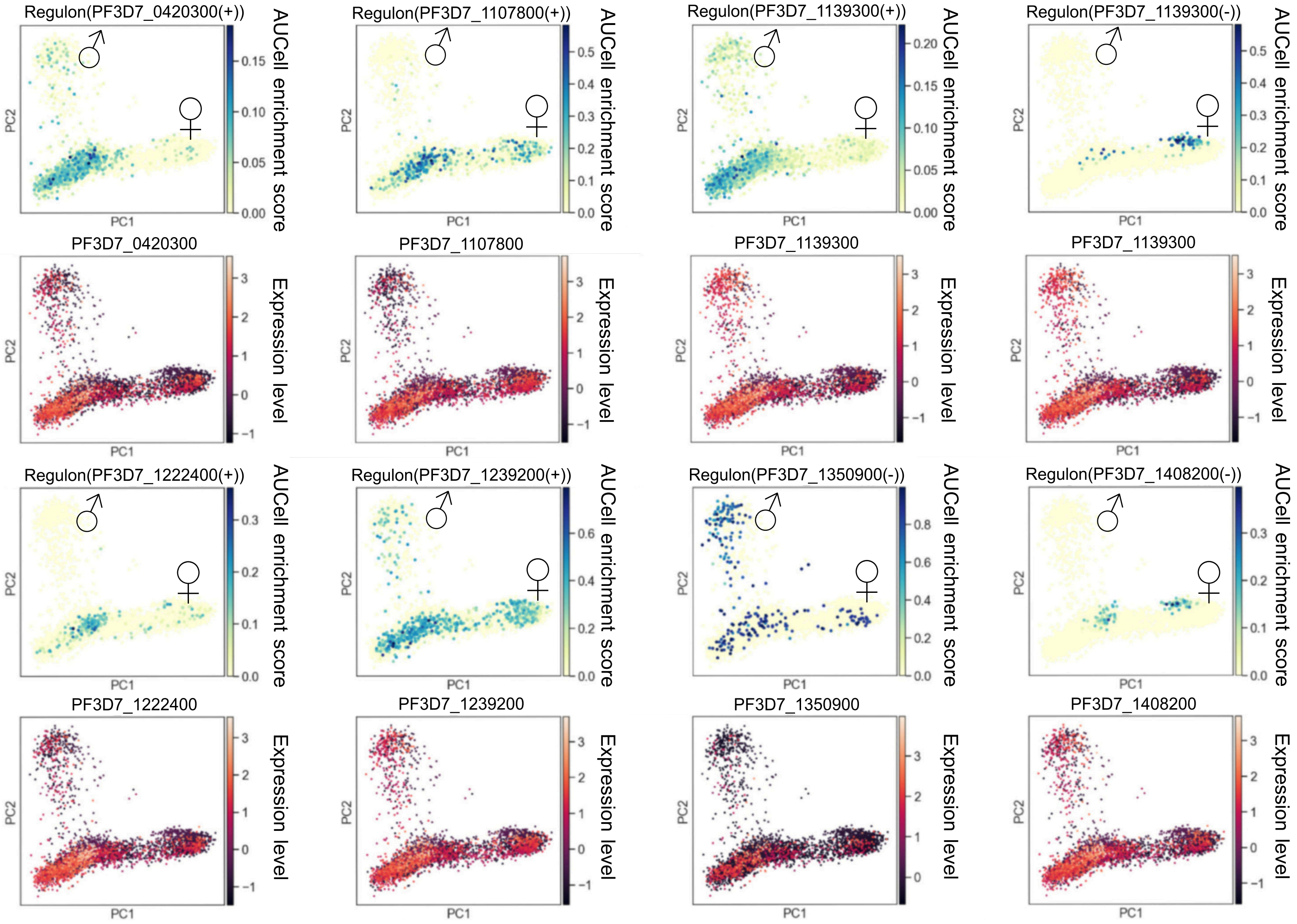

B

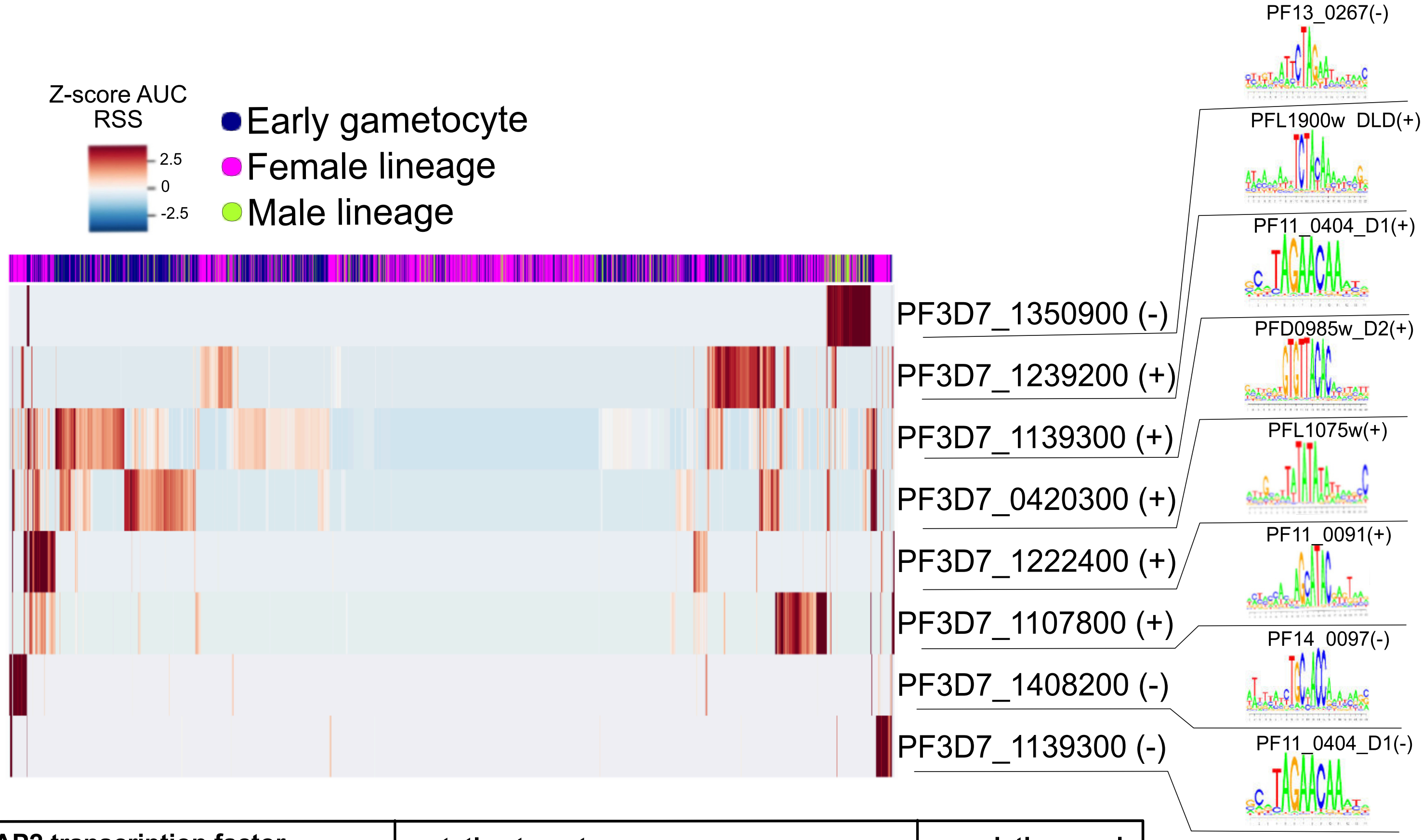

C

ApiAP2 transcription factor		putative target gene	regulation mode
PF3D7_1222400	Mod B	PF3D7_1021600 (DPA), PF3D7_0903700 (alpha tubulin 1)	activating
PF3D7_1107800	Mod B	PF3D7_0903700 (alpha tubulin 1), PF3D7_0204900 (COQ5)	activating
PF3D7_1239200	Mod C	PF3D7_0727600, PF3D7_0219100	activating
PF3D7_1408200-AP2-G2	Mod E	PF3D7_0306200 (AHA1), PF3D7_0209500	repressing
PF3D7_1350900-AP2-O4	Mod D	PF3D7_0604100 (SIP2)	repressing

Supplementary Figure 9: Implementation of Single-Cell rEgulatory Network Inference and Clustering (SCENIC) to infer transcription factors and potential target genes based on motif enrichment using position weight matrices from Campbell et al 2010

A. ApiAP2 enriched regulons by estimation of regulon specificity score (RSS) and projecting AUCell (Area Under the Curve) motif enrichment scores projected on the PCA to determine sex-specific regulon activity. The first and third rows show the regulon AUC score (colored in yellow to dark blue, where dark blue indicates a high score), and the second and fourth rows depict the corresponding TF expression patterns (colored in dark blue to light red, where light red indicates elevated expression levels). B. Heatmap depicting AUC z-score of regulon specificity scores (RSS). RSS for each cell is generated from the AUC distribution (regulators and their associated enriched motifs), annotated across cell class, the color scale represents red (high AUC score) to blue (low AUC score). C. Regulon modules (Mod B to E) mGRN depicting ApiAP2 transcription factor and predicted target genes based on putative motif detection 1.5kb upstream of the annotated transcription start site (TSS).

Supplementary table 1

Gene identity	Gene name	Primer	Sequence
PF3D7_1338800	CPW-WPC family protein	F5-IFW	5-GGGGAGGGCACTAAAAACTCA-3
PF3D7_1338800	CPW-WPC family protein	F5-EFW	5-TGACCATCTTGAGGAACACGG-3
PF3D7_1338800	CPW-WPC family protein	F5-ERW	5-CCTGTCTCTGAATCGACTGGT-3
PF3D7_1466500	Conserved Plasmodium protein, unknown Function	F8-IFW	5-GTGTGTGGATATATTATTTGCTTGTGT-3
PF3D7_1466500	Conserved Plasmodium protein, unknown Function	F8-EFW	5-AGCACCTGTTGAAAATTTATATGAAGA-3
PF3D7_1466500	Conserved Plasmodium protein, unknown Function	F8-ERV	5-TGGACCATTCACATCAATTGTTT-3
PF3D7_0418800	MOLO1domain-containing protein, Putative	F22-IFW	5-ATATGGACGTAAAATGAGTCTATGAT-3
PF3D7_0418800	MOLO1domain-containing protein, Putative	F22-EFW	5-TGTAGTTGGTTTGGCATATGTTG-3
PF3D7_0418800	MOLO1domain-containing protein, Putative	F22-ERV	5-ATTGTCCCTCTGAGCATCCG-3
PF3D7_0924800	Telomeric repeat binding factor 1, Putative	F28-IFW	5-AGCTGACCATGTTCAGAAAAACT-3
PF3D7_0924800	Telomeric repeat binding factor 1, Putative	F28-EFW	5-TGGGAAAAGGAAGAAACAAAATTGC-3
PF3D7_0924800	Telomeric repeat binding factor 1, Putative	F28-ERV	5-ACGATTGGCGAATTTGTGACC-3
PF3D7_1148600	18S ribosomal RNA	18S-FW	5-GTAATTGGAATGATAGGAATTTACAAG-3
PF3D7_1148600	18S ribosomal RNA	18S-ERV	5-TCAACTACGAACGTTTTAACTGCAAC-3

Supplementary Table 1: List of primers used for the rt-qPCR analysis on synchronized gametocyte bulk populations to evaluate the abundance of immature transcripts at different stages of development. Primer sets include one reverse primer and two forward primers, which are positioned either inside the respective intron or in the subsequent exon for selected genes, in order to cross-validate the RNA velocity estimates.

Supplementary table 2

Designation	Codename	Accession Number	Description	Primer F	Primer R
Female Driver 1	FD1	PF3D7_0904200	PH domain-containing protein, putative	ACGAGTAGCCAAATCAAACGA	TGGCCCAGAAATTCCTCAGA
Female Driver 2	FD2	PF3D7_1362600	conserved protein, unknown function	GCGTTGGCTCATAGCTACCAA	GCAAATTGCCCTCTTCCCCT
Female Driver 3	FD3	PF3D7_1338800	CPW-WPC family protein	GTACAGGCTGCGTTCCTCTT	ACACTCCAAGGGCATACACG
Female Driver 4	FD4	PF3D7_1221100.1	conserved Plasmodium protein, unknown function	TGTAATTACCCTGAACAAGCCAA	TGAGATGTTGCACTGTGCTT
Female Driver 5	FD5	PF3D7_1466500	conserved protein, unknown function	TCTGCTTCAGAGAACAAATGACA	ACACCTGCCATCTTCTTAATGGT
Female Driver 6	FD6	PF3D7_1207700	41-3 protein	TCCGTTACCACAAGTTTCTGC	ACTTTGCCATGGACAAGGATT
Female Driver 7	FD7	PF3D7_1005200	conserved Plasmodium protein, unknown function	TGTATGAACAGTCAGGTGAAAAATC	AGCCATTATCTTTTTAGCTTTCGCT
Female Driver 8	FD8	PF3D7_0922300	conserved Plasmodium protein, unknown function	CGGCAAAAGATAGCTGTCGG	ACCACAGCTTTCAGCATCGT
Female Driver 9	FD9	PF3D7_1222800	conserved Plasmodium protein, unknown function	AGGTTGAAATTGCAGAGCAAAAG	AGATCATTGTTCACATTTCTGTCTT
Male Driver 1	MD1	PF3D7_1215500	conserved Plasmodium protein, unknown function	CAATCTTGATGAAGAAACAACAAACAT	TGAGGAAATAAAATCGTTCAGCAAA
Male Driver 2	MD2	PF3D7_0322800	conserved Plasmodium protein, unknown function	TGAACAGTTATTGAAAGCAAAAGAATT	AGCATATGAGCTTCGTCTTACAT
Male Driver 3	MD3	PF3D7_1462500	conserved Plasmodium protein, unknown function	TGGACAATTGAGCGAAGAAGA	AGGACAGCCGTATTAAATCTTTCG
Male Driver 4	MD4	PF3D7_0930200	leucine-rich repeat protein	AGCGTCATTAAAAGAATTGAAGCCA	AAGTTTCTTCCTCTTTATCTTCAACA
Male Driver 5	MD5	PF3D7_1023600	conserved protein, unknown function	ACAGAGGAAAATAGCCAACTTGTG	TCATTTGCTTCCTCCTTTCTTGC
Male Driver 6	MD6	PF3D7_1325200	lactate dehydrogenase, putative	ATTTGTGGAATGGCGGGTGT	TCTTGGAAGGGGGACCATCT
Male Driver 7	MD7	PF3D7_1010400	MORN repeat protein, putative	CAGGCCTAGGTACCTGCATA	ACCATGCTCATACGTGCCTTT
Male Driver 8	MD8	PF3D7_1444200	EF-hand calcium-binding domain-containing protein, putative	GCAAGTACCTAGTGACCTCTACA	TCTACTCCCGATTCGTCTTCA
Male Driver 10	MD10	PF3D7_1456400	EF-hand calcium-binding domain-containing protein, putative	TGAGATAGACACTTCAGAAGACAA	AGTTCGTCAAGCAGTTGGAA
Male Marker 1	MM1	PF3D7_1477700	conserved protein, unknown function	GAGAGGAATTAAATGCTGTTCTTAGAG	ATCAACCATCCCATCCCTATTG
Male Marker 2	MM2	PF3D7_1438800	male development protein MD1	CAATCCAATGATAATAACCACAAAGGAG	TCCTTCCTAATTTCATTGTCCTGAA
Female Marker 1	FM1	PF3D7_1447600	conserved protein, unknown function	CGAAACAGGATGTGGATGGATAG	GCCACAACCACAGGTATCAA
Female Marker 2	FM2	PF3D7_1031000	ookinete surface protein P25	CCATGTGGAGATTTTTCCAAATGTA	CATTTACCGTTACCACAAGTTACATTC
18s rRNA	18S	PF3D7_0112300	18S ribosomal RNA	TCCGATAACGAACGAGATCTTAAC	ATGTATAGTTACCTATGTTCAATTTCA

Supplementary Table 2: List of primers used for bulk and single-cell rt-qPCR analysis, targeting different gametocyte developmental stages for cross-validation of sex-specific expression of the putative male (MD) and female (FD) driver genes. The table includes gene descriptions from PlasmoDB.

Supplementary table 3

Gene_name	Gene_id	Description	*P. gaboni* orthologs
maledriver1	PF3D7_1215500	conserved Plasmodium protein, unknown function	PGSY75_1215500
maledriver2	PF3D7_0322800	conserved Plasmodium protein, unknown function	PGSY75_0322800
maledriver3	PF3D7_1307400	conserved Plasmodium protein, unknown function	PGSY75_1307400
maledriver4	PF3D7_0927400	conserved Plasmodium protein, unknown function	PGSY75_0927400
maledriver5	PF3D7_1468000	WD repeat-containing protein, putative	PGSY75_1468000
maledriver6	PF3D7_1146700	kinesin-X4, putative	PGSY75_0014600
maledriver7	PF3D7_1462500	conserved Plasmodium protein, unknown function	PGSY75_1462500
maledriver8	PF3D7_0930200	leucine-rich repeat protein	PGSY75_0930200
maledriver9	PF3D7_1023600	conserved protein, unknown function	PGSY75_1023600
maledriver10	PF3D7_0922000	dynein intermediate chain, putative	PGSY75_0922000
maledriver11	PF3D7_1325200	lactate dehydrogenase, putative	PGSY75_1325200
maledriver12	PF3D7_1010400	MORN repeat protein, putative	PGSY75_1010400
maledriver13	PF3D7_1444200	EF-hand calcium-binding domain-containing protein, putative	PGSY75_1444200
maledriver14	PF3D7_1235800	peptidase, putative	PGSY75_1235800
maledriver15	PF3D7_1440600	protein SOC3, putative	PGSY75_1440600
maledriver16	PF3D7_1464800	EF-hand calcium-binding domain-containing protein, putative	PGSY75_1464800
maledriver17	PF3D7_1127700	conserved Plasmodium protein, unknown function	PGSY75_1127700
maledriver18	PF3D7_1456400	conserved Plasmodium protein, unknown function	PGSY75_1456400
maledriver19	PF3D7_1135600	condensin-2 complex subunit D3, putative	PGSY75_1135600
maledriver20	PF3D7_1144200	conserved Plasmodium protein, unknown function	PGSY75_1144200
femaledriver1	PF3D7_0904200	PH domain-containing protein, putative	PGSY75_0904200
femaledriver2	PF3D7_1362600	conserved protein, unknown function	PGSY75_1362600
femaledriver3	PF3D7_1338800	CPW-WPC family protein	PGSY75_1338800
femaledriver4	PF3D7_1221100.1	conserved Plasmodium protein, unknown function	PGSY75_1221100
femaledriver5	PF3D7_1466500	conserved Plasmodium protein, unknown function	PGSY75_1466500
femaledriver6	PF3D7_0303900	phosphatidylethanolamine-binding protein, putative	PGSY75_0303900
femaledriver7	PF3D7_1207700	41-3 protein	PGSY75_1207700
femaledriver8	PF3D7_0306200	activator of Hsp90 ATPase	PGSY75_0306200
femaledriver9	PF3D7_1005200	conserved Plasmodium protein, unknown function	PGSY75_1005200
femaledriver10	PF3D7_0922300	conserved protein, unknown function	PGSY75_0922300
femaledriver11	PF3D7_1222800	conserved Plasmodium protein, unknown function	PGSY75_1222800
femaledriver12	PF3D7_1214800	conserved Plasmodium protein, unknown function	PGSY75_1214800
femaledriver13	PF3D7_0903800	LCCL domain-containing protein	PGSY75_0903800
femaledriver14	PF3D7_0920300	conserved Plasmodium protein, unknown function	PGSY75_0920300
femaledriver15	PF3D7_1207300	LIMP protein, putative	PGSY75_1207300
femaledriver16	PF3D7_1322900	conserved protein, unknown function	PGSY75_1322900
femaledriver17	PF3D7_1119200	conserved protein, unknown function	PGSY75_1119200
femaledriver18	PF3D7_0504500	MOLO1 domain-containing protein, putative	PGSY75_0504500
femaledriver19	PF3D7_1146100	PH-like domain-containing protein, putative	PGSY75_1146100
femaledriver20	PF3D7_0719100	ATP synthase F0 subunit a-like protein, putative	PGSY75_0719100

Supplementary Table 3: List of the top 20 predicted male and female driver genes selected for dN/dS analysis, including *P. falciparum* gene accession numbers as well as the gene orthologs in *P. gaboni*.
