# Supplementary material for: Single-cell transcriptomics reveal transcriptional programs underlying male and female cell fate during Plasmodium falciparum gametocytogenesis
Source: Nat Commun. 2024 Aug 26;15:7177. doi: 10.1038/s41467-024-51201-3 (PMC11347709; doi:10.1038/s41467-024-51201-3)
Supplement: Supplementary file 3 — Description of Additional Supplementary Files [file 41467_2024_51201_MOESM3_ESM.docx]

**Description of Additional Supplementary Files**

1. **Supplementary Data 1**: Meta data associated with the single cell barcodes. After data processing, we included all values from the different analyses used in the study. The data is presented in columns with titles across each barcode, to indicate each respective analysis.
2. **Supplementary Data 2**: Data associated with Figure 1C. The table shows results from the Wilcoxon-rank sum test for differentially expressed genes across clusters. Group (left, column 1) indicate the clusters from the Louvain clustering, followed by the gene Accession number (column 2). Score, indicates the ranking score of the gene in each cluster (column 3). Logfold changes, include the changes in expression for each gene/cluster (column 4). pvals, are the p-values of the rank test (column 5). pval_adj, includes the Benjamini-Hochberg (FDR) corrected p-values (column 6).
3. **Supplementary Data 3**: Data associated with Figure 2E. The table shows results from using Wilcoxon-rank sum test to define differentially expressed genes across annotated; early gametocytes, male gametocytes and female gametocytes. The columns represent gene names and result values associated with Supplementary data 2.
4. **Supplementary Data 4**: Excel file containing data associated with Figure 4. The file includes genome locations of the predicted male and female driver genes and specifies the presence of a 3’ intron annotation in PlasmoDB, along with validations of sequence coverage of the 3’ flanking intron, in the 10X sequencing data, for each listed gene (sheets 1 and 3). Additionally, the file contains GO terms for the top 50 putative male and female driver genes (sheets 2 and 4).
5. **Supplementary Data 5**: Excel file containing data associated with Figure 5. Data from the pySCENIC analyses used to infer the gene regulatory networks, which are based on ApiAP2 binding motif predictions, including data from Campbell et al 2010 (sheet 1). In addition, results from the GO term analysis of predicted target genes for each regulator found in the analysis are included (sheet 2).
