# Supplementary material for: Single-cell transcriptomics reveal transcriptional programs underlying male and female cell fate during Plasmodium falciparum gametocytogenesis
Source: Nat Commun. 2024 Aug 26;15:7177. doi: 10.1038/s41467-024-51201-3 (PMC11347709; doi:10.1038/s41467-024-51201-3)
Supplement: Supplementary file 9 — Reporting Summary [file 41467_2024_51201_MOESM9_ESM.pdf]

nature portfolio

Corresponding author(s): Johan Ankarklev, Mubasher Mohammed

Last updated by author(s): Aug 1, 2024

Reporting Summary

Nature Portfolio wishes to improve the reproducibility of the work that we publish. This form provides structure for consistency and transparency in reporting. For further information on Nature Portfolio policies, see our Editorial Policies and the Editorial Policy Checklist.

Statistics

For all statistical analyses, confirm that the following items are present in the figure legend, table legend, main text, or Methods section.

n/a	Confirmed	
☐	☒	The exact sample size (*n*) for each experimental group/condition, given as a discrete number and unit of measurement
☐	☒	A statement on whether measurements were taken from distinct samples or whether the same sample was measured repeatedly
☐	☒	The statistical test(s) used AND whether they are one- or two-sided<br>*Only common tests should be described solely by name; describe more complex techniques in the Methods section.*
☒	☐	A description of all covariates tested
☐	☒	A description of any assumptions or corrections, such as tests of normality and adjustment for multiple comparisons
☐	☒	A full description of the statistical parameters including central tendency (e.g. means) or other basic estimates (e.g. regression coefficient) AND variation (e.g. standard deviation) or associated estimates of uncertainty (e.g. confidence intervals)
☐	☒	For null hypothesis testing, the test statistic (e.g. *F*, *t*, *r*) with confidence intervals, effect sizes, degrees of freedom and *P* value noted<br>*Give P values as exact values whenever suitable.*
☒	☐	For Bayesian analysis, information on the choice of priors and Markov chain Monte Carlo settings
☐	☒	For hierarchical and complex designs, identification of the appropriate level for tests and full reporting of outcomes
☐	☒	Estimates of effect sizes (e.g. Cohen's *d*, Pearson's *r*), indicating how they were calculated

Our web collection on statistics for biologists contains articles on many of the points above.

Software and code

Policy information about availability of computer code

Data collection	Raw data was processed using the open source Cellranger pipeline (version 5.0.1)
Data analysis	scRNA-seq data was analyzed using the following softwares and packages: ScanPy pipeline (V 1.9.1), Monocle 2 (V 2.4.0), Velocyto CLI (v.0.17.17), scVelo package (v1.0.6), pySCENIC protocol (v 012.1), Stringtie2 (v 2.2.1), IGV web app (v 1.12.8), Python (V 3.8.12), Create cisTarget databases Github; https://github.com/aertslab/create_cisTarget_databases.git.<br><br>Code availility statement<br>No new algorithms were developed for this manuscript. Code used for analysis is available in an online repository :https://github.com/ANKARKLEVLAB/Single-cell-P.f-gametocyte

For manuscripts utilizing custom algorithms or software that are central to the research but not yet described in published literature, software must be made available to editors and reviewers. We strongly encourage code deposition in a community repository (e.g. GitHub). See the Nature Portfolio guidelines for submitting code & software for further information.

Data

Policy information about availability of data

All manuscripts must include a data availability statement. This statement should provide the following information, where applicable:

- Accession codes, unique identifiers, or web links for publicly available datasets
- A description of any restrictions on data availability
- For clinical datasets or third party data, please ensure that the statement adheres to our policy

Raw sequencing datasets can be found in Gene expression Omnibus GEO accession number GSE226145 and The raw processed expression matrices, metadata, custom tracks (json), and cis-target motif databases are available via [https://zenodo.org/deposit/7652581]. the data can be interactively visualized at [https://mubasher-mohammed.shinyapps.io/mohammedetal/].

Research involving human participants, their data, or biological material

Policy information about studies with human participants or human data. See also policy information about sex, gender (identity/presentation), and sexual orientation and race, ethnicity and racism.

Reporting on sex and gender	*Use the terms sex (biological attribute) and gender (shaped by social and cultural circumstances) carefully in order to avoid confusing both terms. Indicate if findings apply to only one sex or gender; describe whether sex and gender were considered in study design; whether sex and/or gender was determined based on self-reporting or assigned and methods used.*<br>*Provide in the source data disaggregated sex and gender data, where this information has been collected, and if consent has been obtained for sharing of individual-level data; provide overall numbers in this Reporting Summary. Please state if this information has not been collected.*<br>*Report sex- and gender-based analyses where performed, justify reasons for lack of sex- and gender-based analysis.*
Reporting on race, ethnicity, or other socially relevant groupings	*Please specify the socially constructed or socially relevant categorization variable(s) used in your manuscript and explain why they were used. Please note that such variables should not be used as proxies for other socially constructed/relevant variables (for example, race or ethnicity should not be used as a proxy for socioeconomic status).*<br>*Provide clear definitions of the relevant terms used, how they were provided (by the participants/respondents, the researchers, or third parties), and the method(s) used to classify people into the different categories (e.g. self-report, census or administrative data, social media data, etc.)*<br>*Please provide details about how you controlled for confounding variables in your analyses.*
Population characteristics	*Describe the covariate-relevant population characteristics of the human research participants (e.g. age, genotypic information, past and current diagnosis and treatment categories). If you filled out the behavioural & social sciences study design questions and have nothing to add here, write "See above."*
Recruitment	*Describe how participants were recruited. Outline any potential self-selection bias or other biases that may be present and how these are likely to impact results.*
Ethics oversight	*Identify the organization(s) that approved the study protocol.*

Note that full information on the approval of the study protocol must also be provided in the manuscript.

Field-specific reporting

Please select the one below that is the best fit for your research. If you are not sure, read the appropriate sections before making your selection.

[x] Life sciences [ ] Behavioural & social sciences [ ] Ecological, evolutionary & environmental sciences

For a reference copy of the document with all sections, see nature.com/documents/nr-reporting-summary-flat.pdf

Life sciences study design

All studies must disclose on these points even when the disclosure is negative.

Sample size	Parasites were sorted at four consecutive time points (Days 1, 3, 5, and 7) to cover the P. falciparum gametocyte developmental period, from early to mature male and female gametocytes. However, the total sample size was calculated empirically based on the standards in the field. We used FACS-based cell sorting to specifically enrich gametocytes based on tdTomato fluorescence, followed by droplet encapsulation on 10X Genomics Chromium Controller flowcells. We sorted approximately 10,000 cells from each time point into individual tubes. The cells were then pooled evenly into a single tube with a final concentration of 40,000 cells in total from the four different timepoints and an approximated 20,000 cells were loaded onto the flowcell. To ensure the reproducibility of the gametocyte enrichment strategy, this method was performed twice in biological duplicates using the pre-defined FACS settings for all the data points collected<br>Single-cell data generated using using 10X Chromium Single Cell 3' reagent kits (V3) was mapped to the P. falciparum reference strain 3D7 version 50 (ASM276v2). Based on our filtering criteria, 4555 single cell transcriptomes passed our cut-off for downstream analysis. The sample size is above the state of the art in the field, e.g. in comparison, Gomes et al, Nature, 2022, utilized 2076 single cell gametocyte transcriptomes for their analyses.

Data exclusions	Single-cell data was filtered based on gene and cell counts to remove low quality cells
Replication	Single cell data (including the four time points) was produced from two biological replicates, both replicates were successful and produced similar data
Randomization	N/A
Blinding	N/A

Reporting for specific materials, systems and methods

We require information from authors about some types of materials, experimental systems and methods used in many studies. Here, indicate whether each material, system or method listed is relevant to your study. If you are not sure if a list item applies to your research, read the appropriate section before selecting a response.

Materials & experimental systems

n/a	Involved in the study
☒	☐ Antibodies
☐	☒ Eukaryotic cell lines
☒	☐ Palaeontology and archaeology
☒	☐ Animals and other organisms
☒	☐ Clinical data
☒	☐ Dual use research of concern
☒	☐ Plants

Methods

n/a	Involved in the study
☒	☐ ChIP-seq
☐	☒ Flow cytometry
☒	☐ MRI-based neuroimaging

Eukaryotic cell lines

Policy information about cell lines and Sex and Gender in Research

Cell line source(s)	We used The Plasmodium falciparum gametocyte-producing NF54 Peg4-tdTomato transgenic cell line described in (McLean et al., 2019).
Authentication	Our lab was part of the study that produced the reporter cell line used in the study.
Mycoplasma contamination	The cell line used has previously been tested for mycoplasma, but was not specifically tested during the course of culturing for this study
Commonly misidentified lines (See ICLAC register)	There are no commonly misidentified lines in this study

Plants

Seed stocks	*Report on the source of all seed stocks or other plant material used. If applicable, state the seed stock centre and catalogue number. If plant specimens were collected from the field, describe the collection location, date and sampling procedures.*
Novel plant genotypes	*Describe the methods by which all novel plant genotypes were produced. This includes those generated by transgenic approaches, gene editing, chemical/radiation-based mutagenesis and hybridization. For transgenic lines, describe the transformation method, the number of independent lines analyzed and the generation upon which experiments were performed. For gene-edited lines, describe the editor used, the endogenous sequence targeted for editing, the targeting guide RNA sequence (if applicable) and how the editor was applied.*
Authentication	*Describe any authentication procedures for each seed stock used or novel genotype generated. Describe any experiments used to assess the effect of a mutation and, where applicable, how potential secondary effects (e.g. second site T-DNA insertions, mosiacism, off-target gene editing) were examined.*

Flow Cytometry

Plots

Confirm that:

- [x] The axis labels state the marker and fluorochrome used (e.g. CD4-FITC).
- [x] The axis scales are clearly visible. Include numbers along axes only for bottom left plot of group (a 'group' is an analysis of identical markers).
- [x] All plots are contour plots with outliers or pseudocolor plots.
- [x] A numerical value for number of cells or percentage (with statistics) is provided.

Methodology

Sample preparation	Samples were stained by adding 2 µg/ml of Hoechst live cell stain 33342 to the gametocyte cultures and incubated for 10 mins prior to cell sorting according to the manufacturer's recommendations.
Instrument	MoFlo Astrios EQ (Beckman Coulter, USA)
Software	Summit v 6.3.1
Cell population abundance	Early to late developing gametocytes (tdTomato positive) represent the targeted population
Gating strategy	Parasites were gated based on level of tdTomato fluorescence expression for gametocytes (red) and Hoechst live cell stain 33342 for detection of DNA to avoid doublets. Cell size was also used as part of the gating strategy to remove any background noice

- [x] Tick this box to confirm that a figure exemplifying the gating strategy is provided in the Supplementary Information.
